# A rare case of hypopituitarism with psychosis

**DOI:** 10.1530/EDM-13-0007

**Published:** 2013-08-30

**Authors:** M Nwokolo, J Fletcher

**Affiliations:** Broomfield Hospital, Mid Essex Hospital TrustCourt Road, Chelmsford, CM1 7ETUK

## Abstract

A 46-year-old woman presented multiple times in a 4-month period with hypotension, sepsis, hypoglycaemia and psychosis. A low random cortisol in combination with her presenting complaint made adrenal insufficiency the likely diagnosis. Fluid resuscitation and i.v. steroid therapy led to clinical improvement; however, a short synacthen test (SST) demonstrated an apparently satisfactory cortisol response. The test was repeated on a later admission and revealed a peak cortisol level of 25 nmol/l (>550 nmol/l). Concurrent treatment with i.v. hydrocortisone had led to a false-negative SST. ACTH was <5 ng/l (>10 ng/l), indicating secondary adrenal failure. We discuss the challenges surrounding the diagnosis of adrenal insufficiency and hypopituitarism, the rare complication of psychosis and a presumptive diagnosis of autoimmune lymphocytic hypophysitis (ALH).

**Learning points:**

- Adrenocortical insufficiency must be considered in the shocked, hypovolaemic and hypoglycaemic patient with electrolyte imbalance. Rapid treatment with fluid resuscitation and i.v. corticosteroids is vital.
- Polymorphic presentations to multiple specialities are common. Generalised myalgia, abdominal pain and delirium are well recognised, psychosis is rare.
- A random cortisol can be taken with baseline bloods. Once the patient is stable, meticulous dynamic testing must follow to confirm the clinical diagnosis.
- The chronic disease progression of ALH is hypothesised to be expansion then atrophy of the pituitary gland resulting in empty sella turcica and hypopituitarism.
- If hypopituitarism is suspected, an ACTH deficiency should be treated prior to commencing thyroxine (T_4_) therapy as unopposed T_4_ may worsen features of cortisol deficiency.

## Background

Adrenocortical insufficiency is a challenging diagnosis as it may manifest as a collection of non-specific symptoms. The patient had presented multiple times over the past decade to gynaecologists, physiotherapists and surgeons with amenorrhoea, weight loss, arthralgia, abdominal pain and vomiting. Clinicians should maintain a high index of suspicion when faced with this clinical picture.

## Case presentation

A forty-six-year-old woman was admitted to our DGH four times in a 4-month period with a background of subclinical hypothyroidism on thyroxine (T_4_) replacement, short stature and gravida 3 para 2^+1^. Clinical presentations included hypoglycaemic collapse, persistent hypotension, sepsis with acute kidney injury and psychosis. The patient had no previous mental health issues; however, during this period, her family members reported a dramatic change in personality and behaviour. Emotional liability, poor short-term memory, unprovoked aggression and delusions were observed. She felt people could read her thoughts, claimed to see dead people and described tactile hallucinations, the sensation of insects crawling on her skin. Though cognition apparently remained intact (abbreviated mini-mental test score 10/10), the patient had very limited insight into her altered behaviour.

## Investigation

**Figure 1:**
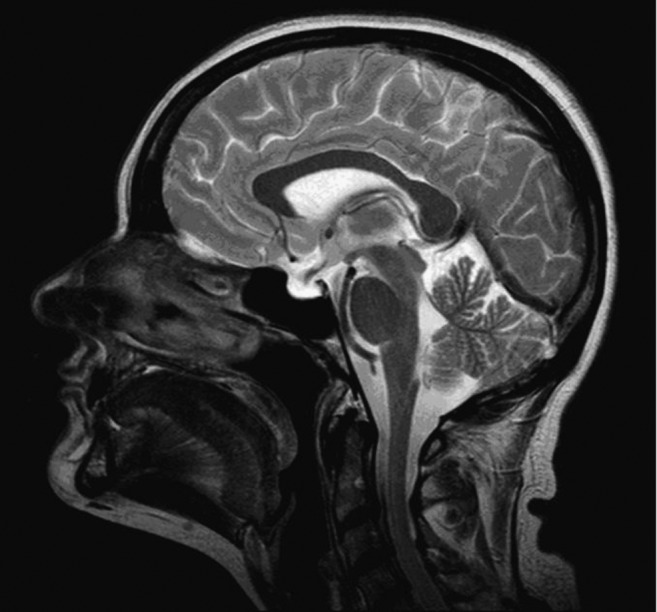
Sagittal T2-weighted MRI head – appearances consistent with empty sella turcica.

For details of the investigation see Table 1, Table 2 and Figure 1.

**Table 1:** Biochemistry with local reference ranges

	**March 2002**	**March 2012**	**April 2012**	**June 2012**
Random cortisol (morning, 140–690 nmol/l; evening 70–350 nmol/l)		11		7
TSH (0.3–5.6 mU/l)	7.59	10.95	9.13	
FT_4_ (6.3–14 pmol/l)	12.5	5.3		
FSH (U/l; follicular 3.8–8.8; mid cycle peak 4.5–22.5; luteal 1.8–5.1; post-menopause >16)	9.4			15.2
LH (U/l; follicular 2.1–11; mid-cycle peak 20–100; luteal 1.2–13; post-menopause >11)	9.0			8.7
Prolactin (0–566 mU/l)	229			343
17β-Oestradiol (post-menopausal <73 pmol/l)	152			<73
17-OH progesterone (follicular 1–8.7 nmol/l; luteal <18 nmol/l)				1.4
GH (μg/l)				0.8
Glucose (fasting 3.6–6.1 mmol/l)	4.9	4.4	3.1	2.5
C-peptide (pmol/l)				<94 pmol/l^a^
Insulin (pmol/l)				<10 pmol/l^a^
β-Hydroxybutyrate				1260 μmol/l^a^
Na (133–146 mmol/l)		128	132	136
K (3.5–5.3 mmol/l)		3.1	4.3	4.0
CRP (0–7.5 mg/l)		261	500	85.3
Creatinine (39–91 μmol/l)		232	340	153

a Results consistent with ketotic hypoinsulinaemia, excludes insulinoma.

**Table 2:** Short synacthen test (SST). Adrenal insufficiency was suspected; however, the patient's initial SST apparently demonstrated a satisfactory cortisol response. Concurrent treatment with i.v. hydrocortisone had led to a false-negative SST. Repeat SST confirmed the clinical diagnosis. Normal response with 0900 h test: stimulated plasma cortisol >550 nmol/l with incremental rise >170 nmol/l. If impaired cortisol response and ACTH >200 ng/l demonstrates primary adrenal failure, ACTH <10 ng/l indicates secondary adrenal failure (Endocrinology Handbook 2010 Imperial College Endocrine Unit)

	**March 2012**	**June 2012**
Cortisol (nmol/l)		
0 min	956	7
30 min	749	9
60 min	771	25
ACTH (ng/l)		
0 min	–	<5

## Treatment

The patient was managed with i.v. fluid resuscitation, broad-spectrum antibiotics and i.v. hydrocortisone 100 mg q.d.s. with rapid clinical improvement.

## Outcome and follow-up

The patient was commenced on long-term hydrocortisone replacement therapy: 10, 5 and 5 mg daily. Prior to her initial admission, she had been commenced on 50 μg T_4_ in the community. This was gradually titrated up to 150 μg over the 4 months.

At 3-month follow-up, the patient reported that her long-standing joint and abdominal pains had resolved. She was menstruating again and there had been no further episodes of psychosis. Her main concern was the 10 kg weight gain since commencing steroid replacement. In response, her hydrocortisone dose was reduced to 10 and 5 mg daily and she remains under 12-month follow-up.

## Discussion

We report a case of hypopituitarism presenting as acute adrenal failure with frank psychosis and empty sella turcica. As symptoms originally developed *post partum* on a background of thyroid hormone deficiency, subclinical autoimmune lymphocytic hypophysitis (ALH) was considered as a unifying diagnosis.

Autoimmune pituitary disease is associated with other autoimmune conditions, notably thyroid (1). ACTH is usually the first hormone deficit in ALH (2) (3), an uncommon condition seen in peripartum women. Lymphocytic infiltration leads to inflammation and expansion of the pituitary gland (4). This may occur silently many years before presenting acutely with adrenal crisis as presumed in this case. In this context, empty sella turcica is hypothesised to be the result of subclinical hypophysitis and subsequent pituitary atrophy (5) (6). Evidence demonstrates that pituitary masses tend to resolve on imaging at long-term follow-up and antipituitary antibodies are detected in over 10% of patients with autoimmune thyroid disease compared with 1% of controls (7).

Here, we note a moderately raised TSH level (10.95 mU/l) despite a particularly low free T_4_ (5.3 pmol/l). This inappropriately mild response of TSH to an overt T_4_ deficiency has been described in secondary hypothyroidism. The complexities surrounding differentiating primary and secondary hormone deficiency can also be seen in the hypothalamic–pituitary–adrenocortical axis. The short synacthen test (SST) has replaced the insulin tolerance test as first line in the investigation of adrenal insufficiency. Clinicians, however, must remain vigilant as mild, recent or acute secondary adrenal hypofunction may result in an inappropriately normal SST (8). As presented here, a high index of clinical suspicion must be maintained to detect a false-negative SST.

Polymorphic presentation to a range of acute services often occurs with adrenocortical insufficiency. Psychiatric manifestations such as mood and behavioural symptoms are reported; however, frank psychosis is less commonly described (9). We suggest that this psychotic episode was triggered by an acutely stressed state on a background of significant steroid deficiency. The swift resolution of the patient's altered behaviour on replacement therapy supports this.

Rapid treatment is always vital in the context of adrenal crisis or acute pituitary decompensation. Meticulous dynamic testing should follow to confirm the diagnosis.

## Patient consent

Written informed consent was obtained from the patient for publication of this case report.

## Author contribution statement

M Nwokolo, specialty registrar to J Fletcher, assembled the case history and investigations from hospital records, analysed the data, and wrote the paper. J Fletcher, named consultant physician of the patient, selected the case, assessed the patient data, and critically reviewed the paper.
